# Circulating Neutrophils Exhibit an Altered Immune Response in Chronic LUTS: An Image-Based Deep Learning Analysis

**DOI:** 10.1007/s00192-026-06625-4

**Published:** 2026-04-18

**Authors:** Catherine C. Y. Chieng, Alia Khoru Azamira, Natasha S. Y. Liou, Qingyang Kong, Artur Yakimovich, Rajvinder Khasriya, Harry Horsley

**Affiliations:** 1 Centre for Kidney and Bladder Health, University College London, London, UK; 2 Present Address: Pantai Hospital, Kuala Lumpur, Malaysia; 3 EGA Institute for Women’s Health, University College London, London, UK; 4 Eastman Dental Institute, University College London, London, UK; 5 Center for Advanced Systems Understanding (CASUS), Görlitz, Germany; 6 Helmholtz-Zentrum Dresden-Rossendorf (HZDR), Dresden, Germany; 7 Institute of Computer Science, University of Wrocław, Wrocław, Poland

**Keywords:** Commensals, Deep learning, Immune response, Neutrophil, Lower urinary tract symptoms, Urinary tract infection

## Abstract

**Introduction and Hypothesis:**

Chronic lower urinary tract symptoms (LUTS) are characterised by persistent symptoms and elevated urinary leukocyte counts. Much evidence supports an infective aetiology in chronic LUTS, often coined chronic UTI, due to the poor performance of current gold standard tests to detect refractory forms of infection. Hence, we hypothesised that neutrophils from chronic LUTS patients may exhibit an aberrant response which prevents complete disease resolution.

**Methods:**

Freshly isolated circulating neutrophils from seven healthy women (control neutrophils) and four patients (patient neutrophils) were stimulated with commensal *E. coli* or pathogenic *E. coli* in artificial urine or pooled urine from controls (control urine) and patients (patient urine). Live imaging was performed at 37 °C for 15 h. Image sequences were analysed to assess neutrophil viability counts, morphometrics, cell activity and bacterial growth.

**Results:**

Neutrophils from both cohorts were able to recognise a commensal *E. coli* from a pathogenic strain, with cell death occurring faster when stimulated with the latter. However, when stimulated with the same bacterial strain in control urine, patient neutrophils showed a higher level of activity and a delayed rate of death. Without bacterial stimulation, patient neutrophils died faster in patient urine. The delayed response and the shorter survival without bacteria exhibited by patient neutrophils may have implications in chronic LUTS patients, where their neutrophils are unable to effectively clear bacteria or survive for an adequate duration to exert their functions.

**Conclusions:**

Temporal differences in neutrophil response towards bacteria and dysregulation in neutrophil maturation or activation may lead to symptom persistence in chronic LUTS.

**Supplementary Information:**

The online version contains supplementary material available at https://doi.org/10.1007/s00192-026-06625-4

## Introduction

Urinary tract infections (UTI) are responsible for a significant global disease burden with 66% and 190% increase in incidence and associated deaths, respectively, from the years 1990 to 2021. It is four times more prevalent in women than in men, with a projected further 45% increase in total cases by 2050 [1]. A proportion of UTI develops into a refractory disease, which has mostly been attributed to unique bacterial behaviours (such as assuming a filamentous morphology, invading the urothelial cells to form intracellular bacterial communities and establishing quiescent intracellular reservoirs) and ecological shifts in the urobiome suggesting polymicrobial infections [2, 3]. In murine models of UTI, the primary immune cell type recruited in response to infection are neutrophils [4]. As urothelial shedding forms part of the bacterial clearance mechanism, there seems to be an intrinsic proclivity of the bladder to skew towards Th2 response that prioritises epithelial regeneration rather than Th1 response that strives for bacterial clearance [5, 6]. Prior infections also increase mouse susceptibility to recurrence and delay recovery in subsequent UTI episodes due to sustained inflammation [7] or insufficient immune memory development [8]. However, observations gleaned from mouse models are yet to be fully substantiated in humans. Moreover, pronounced differences in the immune constituents between mice and humans [9] may hinder direct translation of these findings [10]. Nevertheless, serum levels of cytokines relevant to neutrophil production and chemotaxis, as well as the expression of cell surface markers indicative of neutrophil extravasation, have been found to be increased in the peripheral blood of recurrent UTI patients [11, 12]. Typical clinical presentations of the chronic LUTS patients recruited in this study have been described previously [13–15]. On average, they had been experiencing LUTS (particularly those connected with pain and voiding) for 6 years and had elevated urinary leukocyte counts. Approximately 12% of standard urine culture tests for these patients were positive using the diagnostic threshold of 10^5^ CFU/mL of a single known pathogen. This is unsurprising, with the discovery of urobiome and independent studies reporting on the low sensitivity of urine culture tests in diagnosing UTI [16]. Upon commencing treatment with antibiotics and urinary antiseptics, there were gradual reduction in LUTS and pyuria in these patients, signifying disease improvement and thereby alluding to a highly probable underlying infectious cause. Therefore, we hypothesised that neutrophils from chronic LUTS patients may exhibit an aberrant response that prevents complete disease resolution, potentially leading to chronicity of symptoms. To study this, we isolated circulating neutrophils from healthy controls and chronic LUTS patients to compare their temporal responses towards in vitro bacterial stimulation in both human and simulated urine.

## Materials and Methods

### Experimental Variable 1: Circulating Neutrophils

Healthy controls (*n* = 7) and chronic LUTS patients (*n* = 4) were recruited in this study. The participants were all female aged over 18 years and had no known co-morbidities which may otherwise influence their normal immune functions. Further details on the patient cohort were provided in Supplementary Table 1. Blood samples were collected in EDTA tubes with informed consent from all the participants. Neutrophils were freshly isolated with anti-human StraightFrom® Whole Blood CD66b MicroBeads (Miltenyi Biotec, Germany) using magnetic-activated cell sorting (autoMACS® Pro Separator, Miltenyi Biotec, Germany).

### Experimental Variable 2: Urine Media

Three urine media were used in this study. ‘Control urine’ and ‘patient urine’ were pooled from five healthy individuals and 20 chronic LUTS patients, respectively. The pooled solutions were sterile-filtered (0.22 μm) and stored in aliquots at −20 °C. ‘Artificial urine’ was a commercially available buffered solution containing urea and creatinine (Sigmatrix Urine Diluent, Sigma-Aldrich, UK). Using urinary dipsticks (Multistix® 8 SG Reagent Strips, Siemens Healthineers, UK), all three urine media were negative for leukocytes, nitrite, protein, ketone and glucose with a specific gravity of 1.005. The pH for control urine and patient urine was 6.5 while it was 6.0 for artificial urine. Patient urine was positive for trace haemolysed blood while control urine and artificial urine were negative.

### Experimental Variable 3: Bacterial Isolates

Two clinical isolates of *Escherichia coli* were used in this study. Putative ‘commensal E. coli’ represented a bladder-resident strain from a healthy female who did not have any lower urinary tract symptoms. Putative ‘pathogenic E. coli’ was isolated from a chronic LUTS patient experiencing symptoms of urgency, voiding and pain. Both bacterial strains were previously isolated from urine by overnight aerobic culture on chromogenic agar (CHROMID® CPS® Elite Agar, bioMérieux, UK) at 37 °C, followed by species identification via MALDI-TOF. The isolates were stored in glycerol stocks at −70 °C and revived via overnight aerobic culture on chromogenic agar at 37 °C.

### Time-Lapse Live Imaging of Neutrophils Stimulated with Bacteria in Different Urine Media

**Fig. 1:**
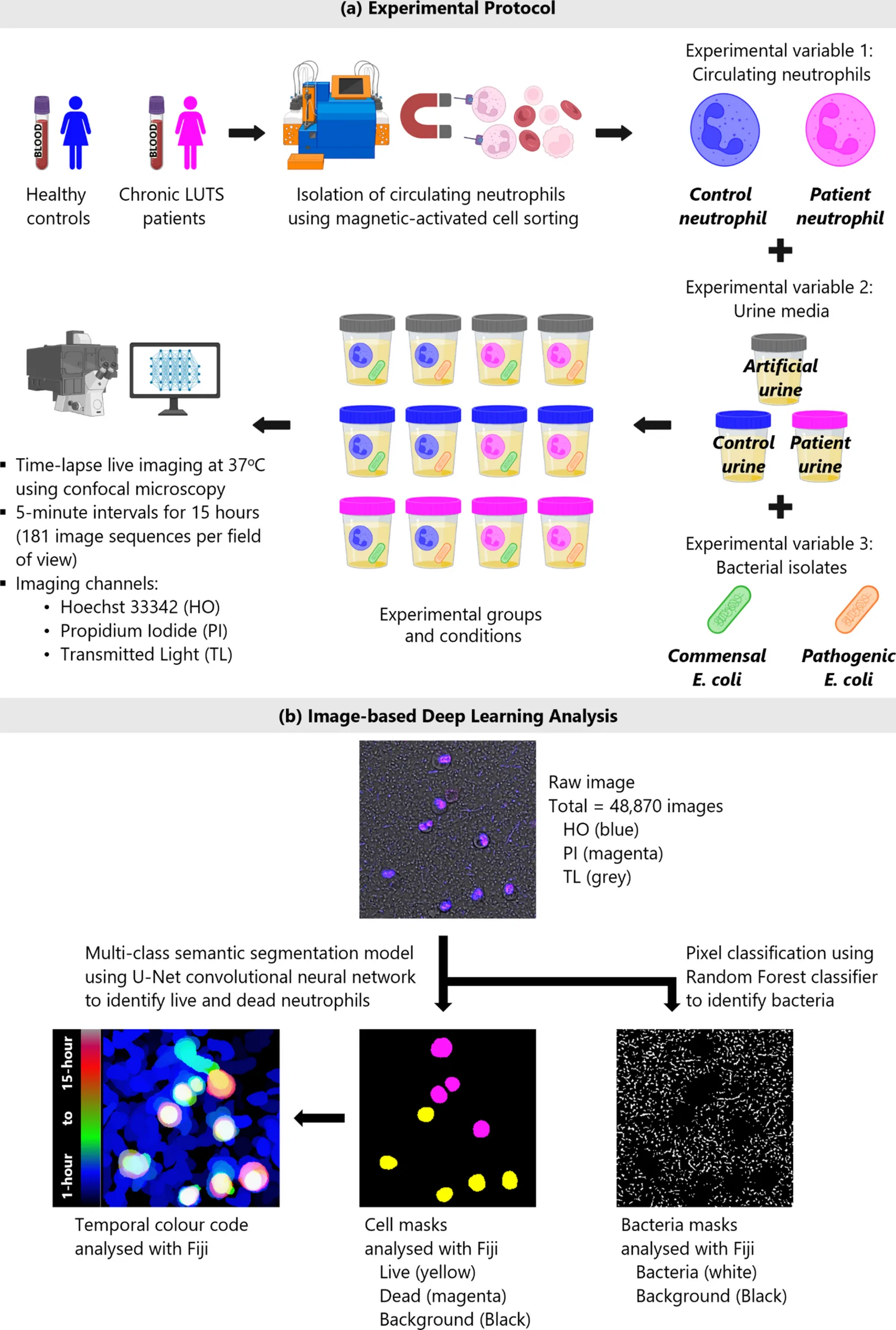
Schematic diagram of the experimental workflow. Time-lapse live imaging of neutrophils stimulated with bacteria in different urine media and image-based deep learning analysis. (**a**) Freshly isolated circulating neutrophils and *E. coli* strains were added at a 1:1 ratio in different urine media for time-lapse live imaging to compare the neutrophil response between healthy controls and chronic LUTS patients. (**b**) The image sequences were analysed with open-source software packages to extract counts and morphometrics for further analysis. Illustrations partly created in https://BioRender.com

All three variables were assembled to produce the experimental groups and conditions shown in Fig. 1. Neutrophils were seeded at 20,000 cells in 300 μl of the three urine media into a microplate (μ-plate 96 well black, ibidi, Germany). The cells were allowed to settle for 30 min before the addition of *E. coli* strains in equal number, resulting in a multiplicity of infection (MOI) of 1. Each well was stained with Hoechst 33342 (1 μg/ml, Thermo Fisher Scientific, UK) and Propidium Iodide (0.5 μg/ml, Thermo Fisher Scientific, UK). Time-lapse live imaging was performed at 37 °C at 5-min intervals for 15 h using a confocal laser scanning microscope equipped with an automated motorised stage (TCS SP8, Leica Microsystems, UK). Images were captured with the addition of Transmitted Light using a 20 × objective in three random fields per well at a resolution of 512 × 512. Experiments with neutrophils (*n* = 3 controls and *n* = 3 chronic LUTS patients) or bacteria alone in the different urine media were also performed using identical settings.

### Image-Based Deep Learning Analysis of Neutrophil Response

**Fig. 2:**
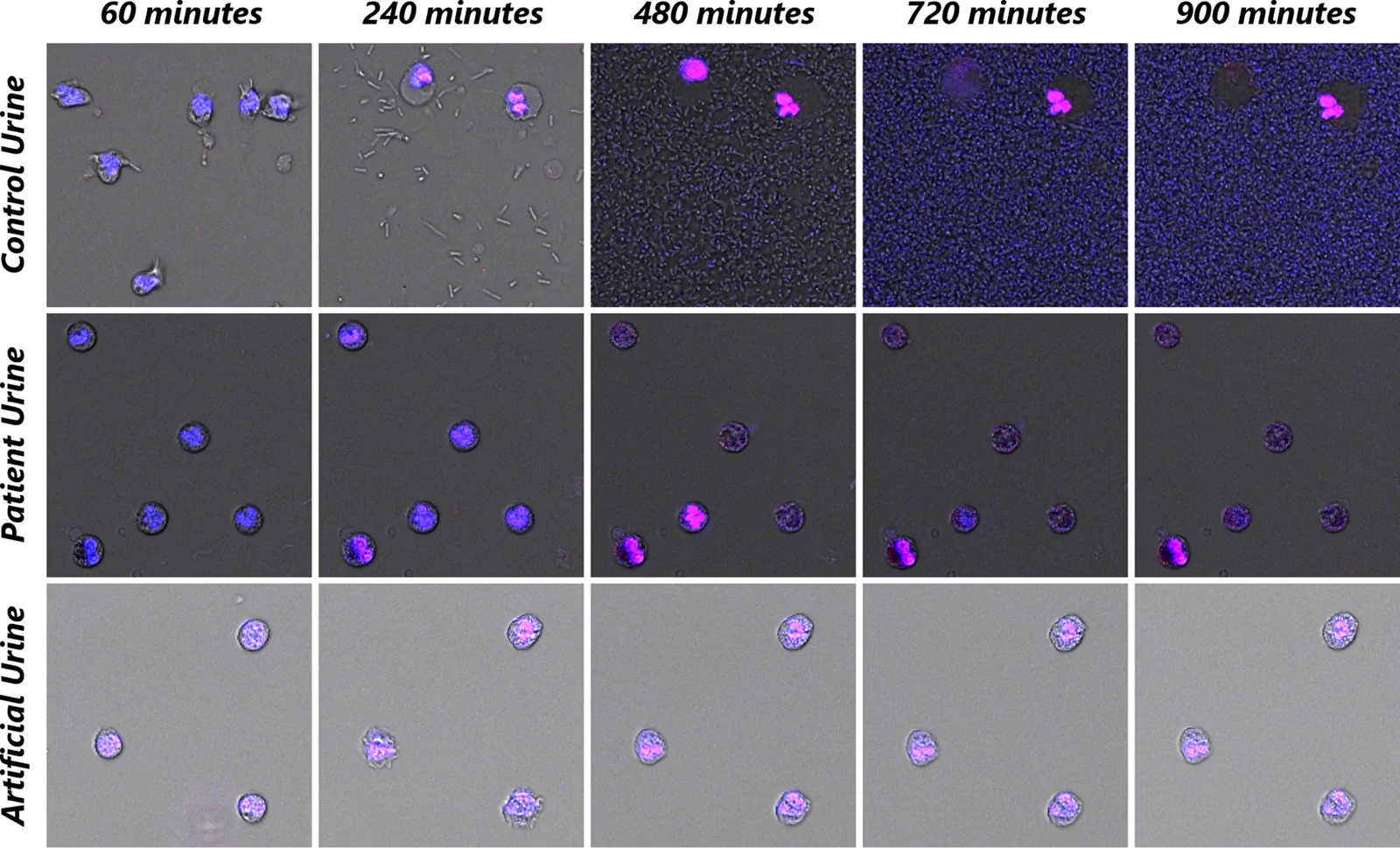
Representative image sequences of neutrophils stimulated with bacteria in different urine media. Neutrophils were stimulated with bacteria at MOI = 1 in different urine media and imaged over time. Live cells were stained blue with Hoechst 33342, while dead cells were stained magenta with Propidium Iodide. The transmitted light channel is shown in grey

**Fig. 3:**
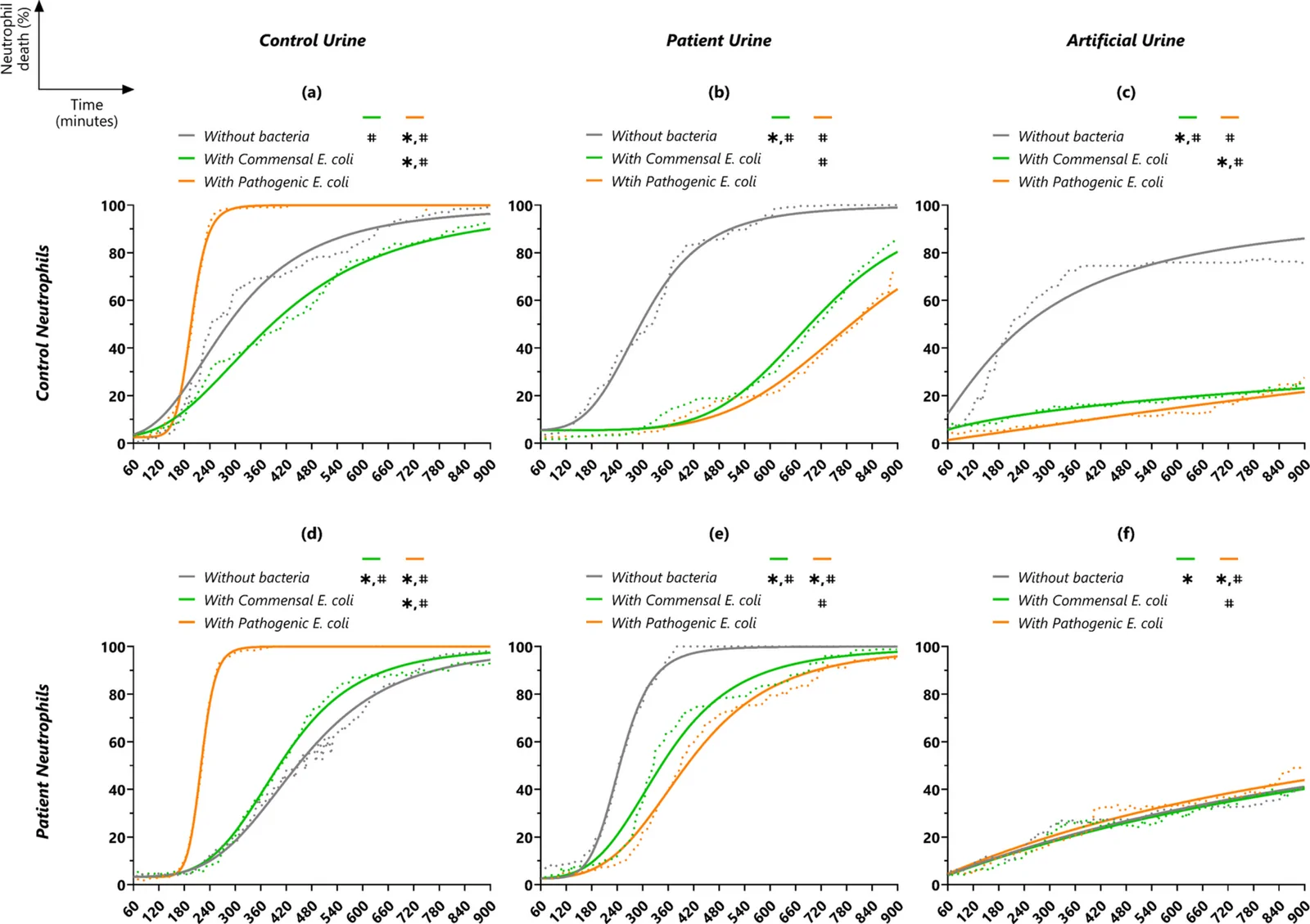
Neutrophil response with or without bacterial stimulation in different urine media. Percentage of neutrophil death in the absence of bacterial stimulation (*n* = 3 per group) and with *E. coli* are shown in control neutrophils (*n* = 7) (**a**-**c**) and patient neutrophils (*n* = 4) (**d**–**f**) in the three urine media. Dotted lines represent mean values, while solid lines show best fit curves analysed by a least squares regression method. Comparisons between best fit curves for rate of neutrophil death (✱) and the time required to achieve half-maximal death (#) are indicated above respective graphs when significantly different at *p* < 0.05 (extra sum-of-squares *F* test)

**Fig. 4:**
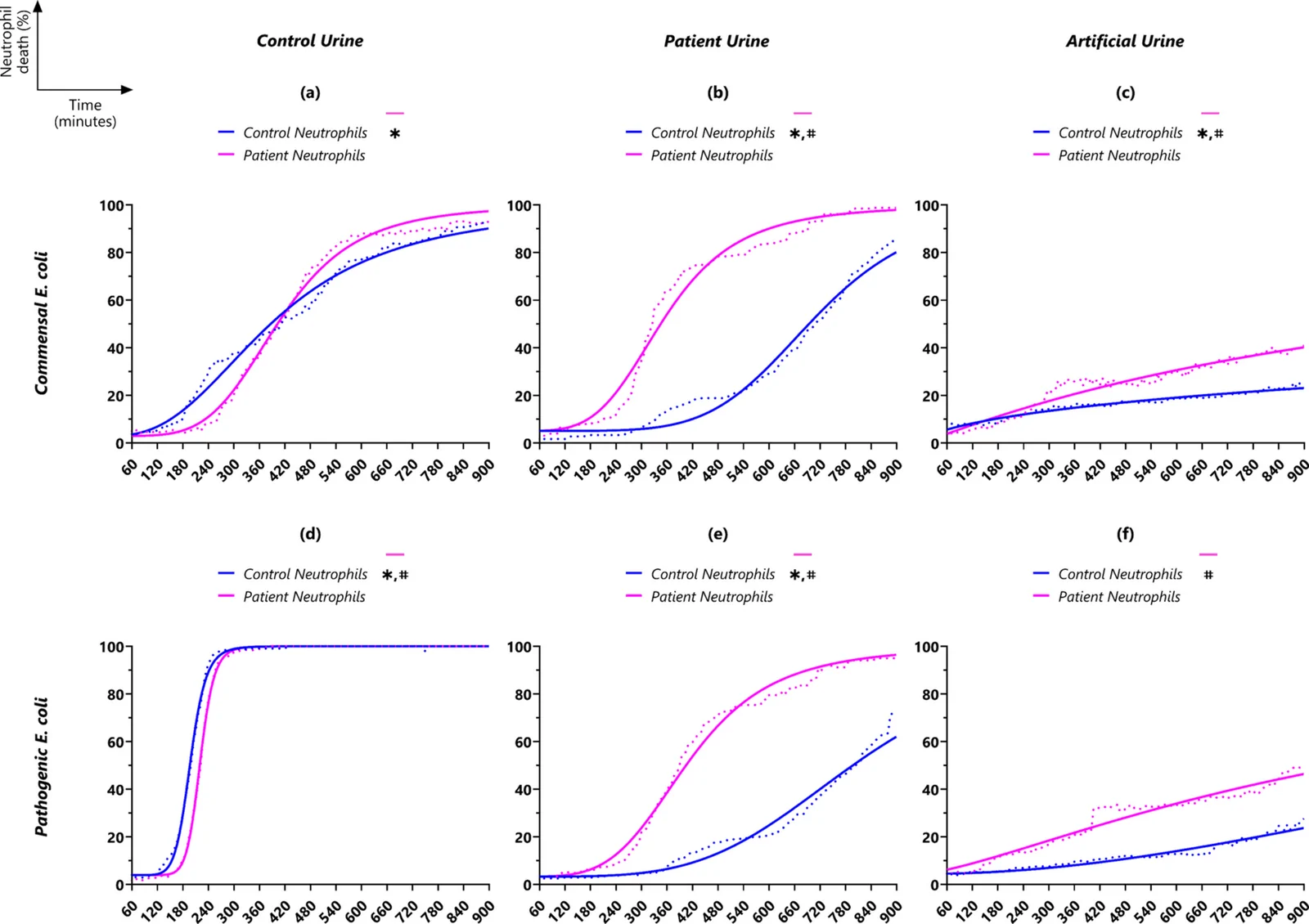
Neutrophil response to bacterial stimulation in different urine media between healthy controls and chronic LUTS patients. Percentage of cell death in neutrophils from healthy controls (*n* = 7) and chronic LUTS patients (*n* = 4) when stimulated with commensal *E. coli* (**a**–**c**) and Pathogenic *E. coli* (**d**–**f**) in the three urine media are presented. Dotted lines represent mean values, while solid lines show best fit curves analysed by a least squares regression method. Comparisons between best fit curves for rate of neutrophil death (✱) and the time required to achieve half-maximal death (#) are indicated above respective graphs when significantly different at *p* < 0.05 (extra sum-of-squares *F* test)

**Fig. 5:**
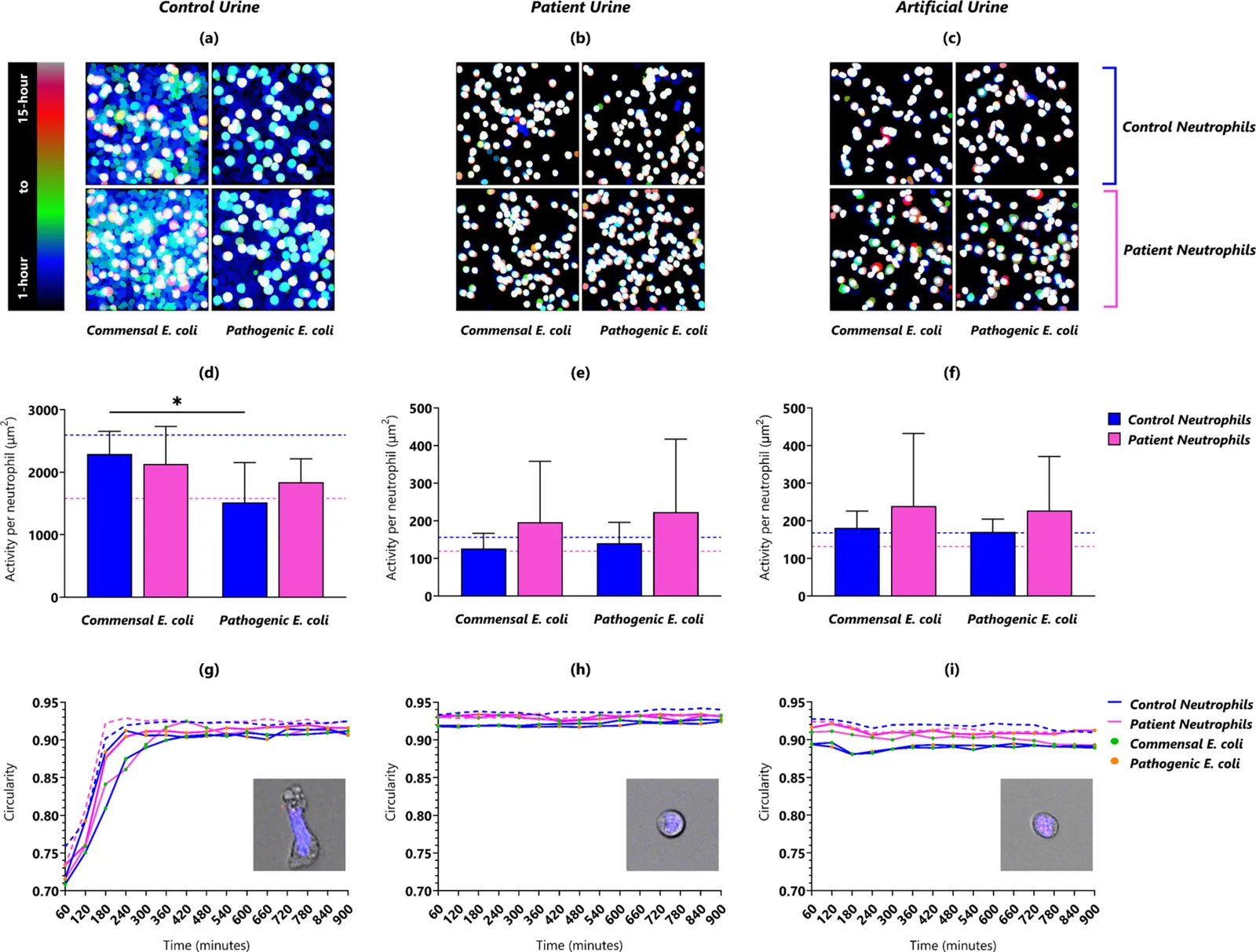
Neutrophil activity in response to bacterial stimulation in different urine media. (**a**–**c**) Overlay images of temporal colour-coded cells to visually represent cumulative activity of neutrophils in response to bacterial stimulation in different urine media. (**d**–**f**) Neutrophil activity was calculated by the area occupied per cell over 15 h. Dotted lines represent baseline activity levels while bar charts show mean values with 95% CI. Significant differences between groups are indicated at *p < 0.05 (one-way ANOVA, *n* = 7 in control, *n* = 4 in patient). (**g**–**h**) Circularity of neutrophils over 15 h (images of a representative cell at the 300-min interval are shown in insets), with dotted lines showing the baseline values, when stimulated with bacteria in the respective urine media

**Fig. 6:**
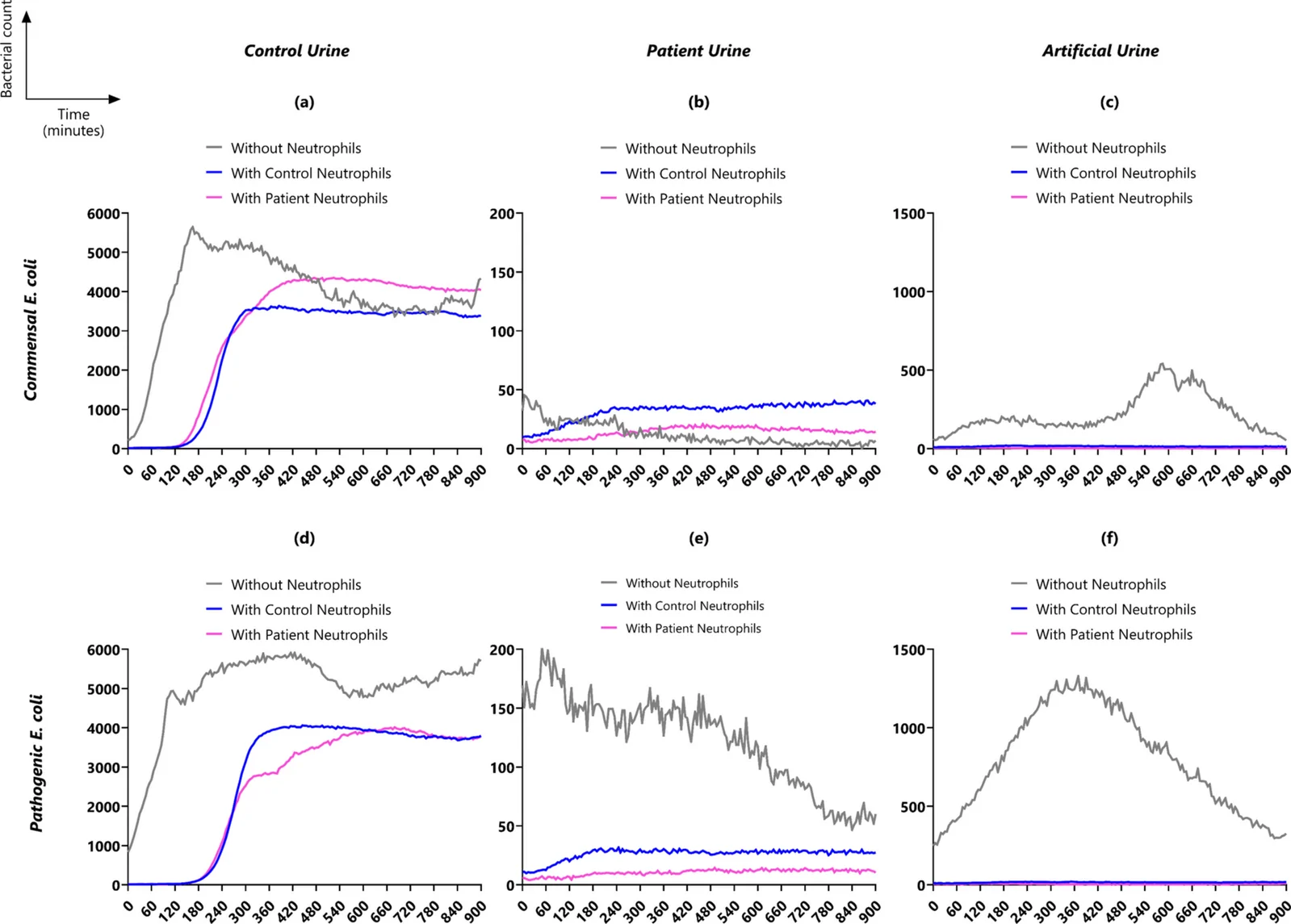
Bacterial growth in the presence of neutrophils in different urine media. Commensal (**a**–**c**) and pathogenic (**d**–**f**) *E. coli* showed growth patterns which were influenced by urine media, with pathogenic *E. coli* showing higher bacterial counts compared to commensal *E. coli*. In the presence of neutrophils, growth inhibition was observed except for commensal *E. coli* in patient urine

Image sequences (examples shown in Fig. 2) were analysed using an open-source deep learning platform, Microscopic Image Analyzer [17]. A multi-class semantic segmentation model was trained to identify image background, live and dead neutrophils. The working model employed a U-Net convolutional neural network with a ResNet50 backbone architecture. Its performance showed an average Dice coefficient of 0.9982 on a test dataset. The resulting cell masks were subsequently processed using Fiji [18] “Analyze Particles” function to extract cell counts, cell area and circularity morphometric (Figs. 3, 4 and 5. The “Temporal-Color Code” function was applied to the image sequences to visualise neutrophil activity (Fig. 5). The pixel classification workflow in ilastik [19], another open source machine learning software utilising random forest classifier for image analysis, was trained to identify bacteria to study the effects of neutrophils and urine media on bacteria over time (Fig. 6). Images from early timepoints were found to fluctuate due to initial focus drift, hence all the images were analysed from 60 min onwards. Statistical analyses to compare between experimental groups and conditions were performed using GraphPad Prism version 10.4.1 for Windows (GraphPad Software, USA).

## Results

### Study Cohorts

The average ages (SD, min–max) were 32.6 (11.6, 22–55) years in controls and 53.5 (18.1, 29–69) years in chronic LUTS patients, with no statistical significance between groups (*p* = 0.0758, Mann–Whitney *U* test). The participants were evaluated with a 39-point LUTS questionnaire [13], with the controls scoring statistically significant lower at a total (SD, min–max) of 1.3 (2.4, 0–6) while the patients scored higher at 6.3 (1.3, 5–8) (*p* = 0.0273, Mann–Whitney *U* test). The average concentration (SD, min–max) of neutrophils (cells/ml) isolated from the blood samples was 8.20 × 10^5^ (3.10 × 10^5^, 2.45 × 10^5^–1.17 × 10^6^) in controls and 9.11 × 10^5^ (2.59 × 10^5^, 7.05 × 10^5^–1.28 × 10^6^) in patients. This was not significantly different between the study groups (*p* > 0.9999, Mann–Whitney *U* test).

### Control and Patient Neutrophil Response was Shaped by Urine Media and Bacterial Stimulation

On the basis of neutrophil activity (Figs. 2, 5d, e and f) and bacterial growth (grey curves in Fig. 6), control urine was deemed to be the most conducive medium for both neutrophils and bacteria, followed by artificial urine and patient urine. Neutrophil response was assessed by rate of cell death and time required to achieve half-maximal death. Without bacterial stimulation (grey curves in Fig. 3), all neutrophils showed the fastest rate of death in patient urine, followed by control urine and artificial urine. With bacterial stimulation, neutrophils showed a significantly slower rate of death in patient urine and artificial urine (Fig. 3b, c and e), except for patient neutrophils in artificial urine (Fig. 3f). In control urine, all neutrophils reached 100% death significantly earlier when stimulated with pathogenic *E. coli* compared to commensal *E. coli* (Fig. 3a and d). Therefore, control neutrophils and patient neutrophils both responded to bacterial stimulation and were able to distinguish between commensal and pathogenic strains.

### Control and Patient Neutrophils Exhibited Distinct Responses Towards the Same Bacterial Strain

Employing the same metrics of neutrophil death rate and time required to achieve half-maximal death, responses exhibited by control neutrophils and patient neutrophils were compared. In patient urine and artificial urine, patient neutrophils died faster than control neutrophils when stimulated with bacteria (Fig. 4b, c, e and f). Conversely, in control urine, patient neutrophils showed a significantly slower rate of death than control neutrophils (Fig. 4a and d). Complete neutrophil death at the end of the 15-h experiment was only observed in neutrophils stimulated with pathogenic *E. coli* in control urine (Fig. 4d). When stimulated with the same bacterial strain, patient neutrophils showed significantly different responses compared to control neutrophils. These results were summarised in Supplementary Table 2.

### Patient Neutrophils Showed Increased Activity Towards Bacteria

Activity level of the neutrophils was visualised via temporal colour-coding (Fig. 5a, b and c) as well as measuring the total area occupied per cell (Fig. 5d, e and f) and cell circularity (Fig. 5g, h and i) over time. Neutrophils were the most active in control urine, followed by artificial urine and patient urine. In control urine, the highest level of activity was observed in the first 3 to 5 h (Fig. 5a and g). At baseline without bacterial stimulation (dotted lines in Fig. 5d, e and f), patient neutrophils were less active than control neutrophils. However, with bacterial stimulation, patient neutrophils showed increased activity, exceeding their baseline and the activity of control neutrophils (bar charts in Fig. 5d, e and f). These results are summarised in Supplementary Table 3.

### Bacterial Growth was Inhibited by Neutrophils

Both commensal *E. coli* and pathogenic *E. coli* showed growth patterns influenced by the type of urine media, with the highest bacterial counts observed in control urine, followed by artificial urine and patient urine (grey curves in Fig. 6). Pathogenic *E. coli* grew in larger numbers compared to commensal *E. coli* in all the urine media. In control urine, in the presence of neutrophils, bacteria showed a longer lag phase prior to commencing exponential growth (Fig. 6a and d), and this rapid growth coincided with neutrophil death (Fig. 4a and d). In patient urine and artificial urine, bacterial growth was relatively static (Fig. 6b, c, e and f) as neutrophils remained viable for longer in these media (Fig. 4b, c, e, and f). Bacteria in the presence of patient neutrophils trended towards a higher degree of inhibition compared to the presence of control neutrophils, particularly in patient urine and artificial urine.

## Discussion

This study examined host circulating neutrophils stimulated with bacteria and showed that chronic LUTS patients exhibited significantly altered temporal responses compared to healthy controls. Mature neutrophils are terminally differentiated cells with characteristic segmented nuclei and cytoplasmic granules, executing antimicrobial mechanisms such as phagocytosis, degranulation, reactive oxygen species production and release of neutrophil extracellular traps [20]. Studies have shown that neutrophils can also perform these effector functions in urine [21, 22]. Healthy human urine is primarily water containing urea, creatinine, electrolytes, proteins, metabolites, cells from the urinary tract and bladder-resident bacteria [10, 23–25]. This study used cell-free, sterile-filtered human urine and found that both neutrophils and bacteria showed the greatest level of activity and growth in control urine. Therefore, responses of control neutrophils in control urine were used as the benchmark from which to compare patient neutrophils when challenged under different experimental conditions. In patient urine and artificial urine, neutrophil activity levels and bacterial growth were lower. As patient urine was pooled from chronic LUTS patients being treated with antibiotics and urinary antiseptics [14], it likely contained drug residues [26] that may have been inhibitory to neutrophils and bacteria. Conversely, the simplistic nature of artificial urine may not have been sufficient to support neutrophil activity or bacterial growth.

Neutrophils express pattern recognition receptors (PRRs) which interact with pathogen-associated molecular patterns (PAMPs) to initiate an immune response [20]. Even though our in vitro experiments lack the complexities of in vivo multicellular interactions, neutrophils from both study cohorts were capable of mounting distinct responses towards commensal and pathogenic *E. coli* strains. Commensals have been shown to work in concert with the immune system to maintain their colonisation and homeostasis in the gastrointestinal tract. Microbiota can dampen the host immune response by regulating the differentiation of effector T cells and Tregs, or by structurally modifying their PAMPs to extenuate inflammation. Commensals also produce short-chain fatty acids and engage PRRs on neutrophils to modulate neutrophil production, activation and function [27–30]. In this study, all neutrophils generally showed a less rapid cell death when stimulated with commensal *E. coli* compared to pathogenic *E. coli* (Fig. 4a and d). A previous study showed that after ingestion of *E. coli*, neutrophils underwent phagocytosis-induced cell death, which was dependent on subsequent respiratory bursts [31]. This accelerated apoptosis is likely advantageous to the host to restrict tissue damage caused by the release of bactericidal compounds from the neutrophils, which are also cytotoxic [32]. In one study involving peripheral neutrophils from healthy controls, urine exerted an opsonic effect on *E. coli* and the percentage of bacteria phagocytosed was at least twofold greater in urine from UTI patients compared to urine from healthy controls [21]. Another study found that peripheral neutrophils from UTI patients showed lower levels of phagocytosis and reactive oxygen intermediates (ROI) production when challenged with *E. coli* [11], though the assays were not performed in urine. In our study, neutrophils from both cohorts showed endpoints approaching or reaching 100% cell death in control urine, but the rate was slower in patient neutrophils. The rates of response from all neutrophils were further reduced in patient urine and artificial urine (Fig. 3), but patient neutrophils showed quicker responses in these media. These varied results, dependent on experimental stimuli, suggest that patient neutrophils were functionally intact, with the main difference being in the rate and timing of response in comparison to control neutrophils.

In this study, patient neutrophils exhibited reduced baseline activity (Fig. 5) which increased upon bacterial stimulation while the inverse was true for control neutrophils. Patient neutrophils also showed a faster rate of death in patient urine at baseline without bacterial stimulation (Fig. 3b and e). This relative inactivity and shorter survival shown by patient neutrophils in patient urine may have implications in bacterial clearance during acute symptom flares in chronic LUTS patients, as the neutrophils are either unable to initiate a response quick enough or persist for an adequate duration of time to efficiently perform their bactericidal roles. In the aforementioned study [11], neutrophils from UTI patients showed higher levels of ROI production and cell surface markers for recruitment and phagocytosis at baseline compared with neutrophils from controls. Of note, expression of CD11c, which is crucial for neutrophil maturation and enhanced effector functions [33], was also higher in patients at baseline. These parameters all increased further when stimulated with *E. coli* to the same levels as the control group [11]. Therefore, we postulated that baseline activity may reflect the degree to which neutrophils are primed for subsequent activation when exposed to a secondary stimulus, or apoptosis [34]. Moreover, since the patient cohort in this study has a chronic disease, the neutrophils may have acquired a longer-lasting, altered functional response to infections due to the development of innate immune memory—a rapidly growing field of study termed “trained immunity” [35]. Nevertheless, despite the neutrophils being less active in patient urine and artificial urine, their presence still inhibited the proliferation of bacteria (Fig. 5). However, this could be due to the simultaneous slow growth of bacteria in these media, hence the low bacterial count could readily be contained by the neutrophils, which did not reach 100% death at the end of these experimental conditions.

One of the limitations of our study was the relatively small sample size. Nonetheless, the application of time-resolved imaging and objective analyses with deep learning, instead of a fixed endpoint assay, was able to demonstrate differences in the neutrophil response kinetics between healthy and chronic LUTS individuals. There was also a slight age disparity between the cohorts, due to the larger age range in patient group. In a cross-sectional study examining different age groups, neutrophil chemotaxis, phagocytosis, anti-tumour activity of NK cells, lymphocyte chemotaxis and proliferation showed decline with age (50–79 years) but were maintained in long-lived adults (90–103 years) when compared to 30–49 years, with lifestyle and diet as influencing factors. Thus, immune decline is not a certainty with chronological age; rather, immune functions provide a clue to biological age [36, 37]. In our study, the higher cell concentration and greater level of activity shown by patient neutrophils upon bacterial stimulation suggested that the cohort differences observed in this study were most likely driven by their respective healthy or chronic infection profiles. For future works, a study with a bigger sample size can be performed to substantiate the universality of the trends seen in this study, with more detailed analysis using neutrophil functional assays and specific cellular markers. Moreover, it would be important to phenotypically and genotypically type the putative commensal and pathogenic *E. coli* and characterise the components of the urine media. This information would allow us to better understand the divergent neutrophil responses toward these bacterial strains and urine environments. It would also be interesting to study a separate group of chronic LUTS patients who have recovered to determine if neutrophil behaviours are reinstated to the levels seen in healthy controls or remain altered upon disease remission. From a clinical perspective, neutrophil response signatures may be useful as a prognostic tool to stratify patients based on disease severity and response to treatment. Furthermore, immunomodulatory therapies targeting neutrophils may be able to complement existing therapies, potentially promoting antibiotic stewardship and efficacy in the treatment of chronic LUTS.

## Conclusion

In conclusion, for the first time, this study revealed significant differences in circulating neutrophil response kinetics between healthy controls and chronic LUTS patients. Patient neutrophils may have functional heterogeneity in exerting bactericidal effects or possible dysregulation in their maturation and activation, which in turn promote the magnitude and chronicity of LUTS. The underlying mechanisms for the altered neutrophil response warrant further investigation to harness neutrophils as potential therapeutic targets in chronic LUTS.

## Supplementary Information

Below is the link to the electronic supplementary material.

Supplementary file1 (DOCX 17 KB)

## Data Availability

The original contributions presented in this study are included in the article. Further inquiries may be directed to the corresponding author.

## Abbreviations

CI: Confidence intervals
Ho: Hoechst 33342
LUTS: Lower urinary tract symptoms
MALDI-TOF: Matrix-assisted laser desorption/ionization time-of flight
min-max: Minimum to maximum
PAMPs: Pathogen-associated molecular patterns
PI: Propidium iodide
PRRs: Pattern recognition receptors
ROI: Reactive oxygen intermediates
SD: Standard deviation
TL: Transmitted light
UTI: Urinary tract infection
