# Supplementary material for: Circulating Neutrophils Exhibit an Altered Immune Response in Chronic LUTS: An Image-Based Deep Learning Analysis
Source: Int Urogynecol J. 2026 Apr 18;37(9):2879–89. doi: 10.1007/s00192-026-06625-4 (PMC13649800; doi:10.1007/s00192-026-06625-4)
Supplement: Supplementary file 1 [file 192_2026_6625_MOESM1_ESM.docx]

**Supplementary Table 1.** Clinical details of patients with chronic lower urinary tract symptoms (LUTS).

| **Sample** | **Age** | **Estimated symptom duration** | **LUTS score (out of 39)** | **Dipstick leukocyte esterase** | **Dipstick nitrite** | **MSU culture*** | **Fresh urinary leukocyte per μL** |
| --- | --- | --- | --- | --- | --- | --- | --- |
| P01 | 29 | 15 years | 5 | Negative | Negative | Negative | 34 |
| P02 | 64 | 7 years | 6 | Negative | Negative | Negative | 0 |
| P03 | 69 | 8 years | 6 | 1+ | Negative | Positive | 26 |
| P04 | 50 | 8 years | 7 | 1+ | Negative | Negative | 4 |

* A positive result is indicated by growth of 10^5^ CFU/mL of a single known pathogen.

**Supplementary Table 2.** Summary of neutrophil death response between healthy control and patient groups in the experimental conditions tested in this study.

| **Urine medium** | **Bacterial strain** | **Control Neutrophil** | **Patient Neutrophil** | **Figure** |
| --- | --- | --- | --- | --- |
| Control Urine | - | Faster | Slower | 3a, 3d |
| Patient Urine | - | Slower | Faster | 3b, 3e |
| Artificial Urine | - | Faster | Slower | 3c, 3f |
| Control Urine | Commensal *E. coli* | Faster and became slower after 420 minutes | Slower and became faster after 420 minutes | 4a |
| Patient Urine | Commensal *E. coli* | Slower | Faster | 4b |
| Artificial Urine | Commensal *E. coli* | Slower | Faster | 4c |
| Control Urine | Pathogenic *E. coli* | Faster | Slower | 4d |
| Patient Urine | Pathogenic *E. coli* | Slower | Faster | 4e |
| Artificial Urine | Pathogenic *E. coli* | Slower | Faster | 4f |

**Supplementary Table 3.** Summary of neutrophil activity response between healthy control and patient groups in the experimental conditions tested in this study.

| **Urine medium** | **Bacterial strain** | **Control Neutrophil** | **Patient Neutrophil** | **Figure** |
| --- | --- | --- | --- | --- |
| Control Urine | - | More active | Less active | 5d |
| Patient Urine | - | More active | Less active | 5e |
| Artificial Urine | - | More active | Less active | 5f |
| Control Urine | Commensal *E. coli* | More active | Less active | 5d |
| Patient Urine | Commensal *E. coli* | Less active | More active | 5e |
| Artificial Urine | Commensal *E. coli* | Less active | More active | 5f |
| Control Urine | Pathogenic *E. coli* | Less active | More active | 5d |
| Patient Urine | Pathogenic *E. coli* | Less active | More active | 5e |
| Artificial Urine | Pathogenic *E. coli* | Less active | More active | 5f |
